# Quality of life after photo-selective vaporization and holmium-laser enucleation of the prostate: 5-year outcomes

**DOI:** 10.1038/s41598-019-44686-2

**Published:** 2019-06-04

**Authors:** Inyoung Sun, Sangjun Yoo, Juhyun Park, Sung Yong Cho, Hyeon Jeong, Hwancheol Son, Seung-June Oh, Jae-Seung Paick, Min Chul Cho

**Affiliations:** 1 Department of Urology, Seoul National University College of Medicine, SMG-SNU Boramae Medical Center, Seoul, 07061 Republic of Korea; 2 Department of Urology, Seoul National University College of Medicine, Seoul National University Hospital, Seoul, 03080 Republic of Korea

**Keywords:** Prostate, Urological manifestations, Quality of life

## Abstract

This study was aimed to compare serial long-term postoperative changes in quality-of-life (QoL) between photoselective-vaporization (PVP) using 120W-High-Performance-System and holmium-laser-enucleation (HoLEP) in benign-prostatic-hyperplasia (BPH) patients and to identify factors influencing the QoL improvement at the short-term, mid-term and long-term follow-up visits after surgery. We analyzed 1,193 patients with a baseline QoL-index ≥2 who underwent PVP (n = 439) or HoLEP (n = 754). Surgical outcomes were serially compared between the two groups at up to 60-months using the International-Prostatic-Symptom-Score (I-PSS), uroflowmetry, and serum PSA. We used logistic regression analysis to identify predictors of QoL improvement (a reduction in the QoL-index ≥50% compared with baseline) at the short-term (12-months), mid-term (36-months), and long term (60-months) follow-up after surgery. In both groups, the QoL-index was decreased throughout the entire follow-up period compared with that at baseline. There were no significant differences in postoperative changes from the baseline QoL-index between the two groups during the 48-month follow-up, except at 60-months. The degree of improvement in QoL at 60-months after HoLEP was greater than that after PVP. A lower baseline storage-symptom-subscore and a higher bladder-outlet-obstruction-index (BOOI) were independent factors influencing QoL improvement at the short-term. No independent factor influences QoL improvement at the mid- or long-term.

## Introduction

Lower urinary tract symptoms (LUTS) caused by benign prostatic hyperplasia (BPH) is known to be a highly prevalent disease with increasing age^1–3^, and it is directly and negatively related to the quality of life (QoL)^2,4^. As expectations for QoL are increasing with increased life expectancy, many elderly men with LUTS become less tolerant^5^ and complain about LUTS, leading to poor QoL, although LUTS due to BPH is not life threatening. Thus, one of the aims of treatment for BPH patients is improving QoL through improving LUTS^6^, and, to improve LUTS and QoL in men with severe LUTS, surgical treatment would be useful^5^.

For decades, standard treatment for BPH was transurethral prostatectomy (TURP)^3,7,8^. Recently, as alternatives for TURP, laser surgeries such as photoselective vaporization of the prostate (PVP) and holmium laser enucleation of the prostate (HoLEP) have been increasingly performed^9^, because of fewer perioperative complications, which are related to perioperative QoL^10^. Specifically, in several studies, PVP and HoLEP were reported to have, at least, noninferior efficacy, less bleeding, and less catheter duration than TURP^11^. Thus, PVP was recommended in patients with a high cardiovascular risk and high bleeding risk^12^; additionally, HoLEP has an advantage regarding hemostasis^13^. As less complications would be expected to lead to better QoL as mentioned above^10^, PVP and HoLEP could be the good option to LUTS patient who pursuit QoL.

To the best of our knowledge, few studies have directly compared postoperative treatment outcomes between PVP and HoLEP, focusing on improvement in QoL during a serial long-term follow-up period, although a few short-term follow-up studies showed no significant differences in the postoperative improvement of QoL during a one-year follow-up after surgery^14,15^. Additionally, few studies have investigated which factors could influence postoperative improvements in QoL after the two laser surgeries. Therefore, the aim of this study was to compare serial long-term postoperative changes in QoL between PVP using a 120-W GreenLight high-performance system (HPS) and HoLEP in patients with BPH and to determine the factors influencing the improvement of QoL at the short-term, mid-term, and long-term follow-up visits after surgery using a serial long-term follow-up database.

## Results

### Baseline characteristics

In comparing the baseline characteristics between the PVP and HoLEP groups, the PVP group had a higher level of serum PSA, a smaller prostate volume, a higher voiding symptom score (VSS), a higher storage symptom score (SSS), a higher total International Prostate Symptom Score (I-PSS), a longer operation time, and greater energy applied during the surgeries than the HoLEP group (Table 1). However, the baseline QoL index was not different between the groups.

**Table 1:** Preoperative and perioperative data of the PVP and HoLEP groups.

	Total (n = 1,193)	PVP (n = 439)	HoLEP (n = 754)	P-value
Mean ± SD or No. pts (%)				
Patient demographics				
Age (yr)	68.5 ± 7.1	68.0 ± 8.0	68.7 ± 6.6	0.093
BMI (kg/m^2^)	24.0 ± 3.0	23.9 ± 3.1	24.1 ± 3.0	0.420
PSA (ng/ml)	4.2 ± 6.0	5.2 ± 8.0	3.6 ± 4.1	<0.001^*^
TPV (ml)	56.8 ± 28.4	51.4 ± 32.0	60.0 ± 25.5	<0.001^*^
Symptom scores				
Total I-PSS	19.7 ± 7.6	20.6 ± 8.1	19.2 ± 7.4	0.003^*^
VSS	11.9 ± 5.2	12.3 ± 5.3	11.7 ± 5.1	0.029^*^
SSS	7.8 ± 3.5	8.3 ± 3.7	7.6 ± 3.4	0.001^*^
QoL index	4.2 ± 1.0	4.3 ± 1.0	4.2 ± 1.0	0.207
Uroflowmetric parameters				
*Q*_max_ (ml/s)	10.4 ± 4.8	10.6 ± 5.5	10.3 ± 4.6	0.548
PVR (ml)	73.1 ± 102.5	83.2 ± 117.3	67.2 ± 92.3	0.018^*^
BVE (%)	73.2 ± 24.1	72.0 ± 24.5	73.9 ± 23.9	0.225
Urodynamic parameters				
FDV (mL)	187.4 ± 82.5	182.1 ± 90.4	190.4 ± 77.5	0.112
MCC (mL)	378.8 ± 117.5	405.0 ± 89.5	365.3 ± 127.5	<0.001^*^
IDC	478 (40.1)	148 (33.7)	330 (43.8)	0.028^*^
BOOI	40.6 ± 27.1	35.4 ± 28.2	43.7 ± 26.1	<0.001^*^
BCI	94.6 ± 30.1	88.7 ± 31.4	98.1 ± 28.8	<0.001^*^
Perioperative data				
Operation time (min)	69.7 ± 37.9	72.1 ± 46.5	68.5 ± 32.5	0.018^*^
Enucleation ratio			0.78 ± 0.47	
Used energy (joules)	102.5 ± 63.5	129.1 ± 90.8	88.7 ± 35.8	<0.001^*^

Note: BMI - body mass index, PSA - prostate specific antigen, TPV – total prostate volume, I-PSS - International Prostatic Symptom Score, VSS - voiding symptom score, SSS - storage symptom score, QoL - quality of life, *Q*_max_ - peak flow rate, PVR - postvoid residual urine volume, BVE - bladder voiding efficiency, FDV – volume on the first desire to void, MCC - maximum cystometric capacity, IDC - involuntary detrusor contraction, BOOI - bladder outlet obstruction index, BCI - bladder contractility index. Bladder outlet obstruction index (Abram-Griffiths number) was defined as detrusor pressure at maximum flow rate (*Q*_max_) − 2 *Q*_max_. Bladder contractility index was defined as detrusor pressure at maximum flow rate (*Q*_max_) + 5 *Q*_max_. Enucleation ratio was defined as enucleated weight of prostatic tissue/prostate volume.

The asterisk (*) indicates a statistically significant difference (Independent t-test or Chi-squared test, p < 0.05).

Regarding the baseline urodynamic data, the HoLEP group showed a smaller postvoid residual urine volume (PVR), a smaller maximum cystometric capacity (MCC), a higher bladder outlet obstruction index (BOOI), a higher bladder contractility index (BCI), and a higher percentage of patients with involuntary detrusor contraction (IDC) than the PVP group (Table 1).

### Serial postoperative outcomes after PVP or HoLEP

**Figure 1:**
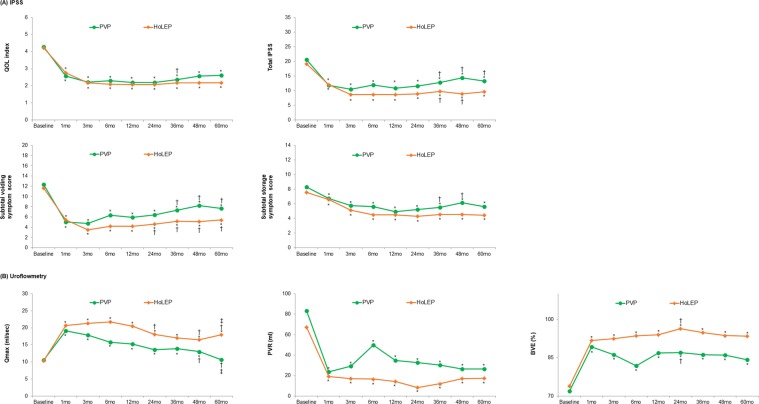
Serial postoperative outcomes after PVP and HoLEP. The asterisk (*) indicates that, at each follow-up visit, significant differences were found from the value at baseline. A dagger (†) and double dagger (‡) indicate that, at each follow-up visit, a significant difference was found from the baseline value at 1 year and 3 years of follow up, respectively (Paired t-test, p < 0.05).

In both the PVP and HoLEP groups, the value of the QoL index at each follow-up visit was significantly decreased during the entire follow-up period after surgery compared with that at the baseline (Fig. 1). Additionally, according to the repeated measures analysis of variance (ANOVA) test to adjust for the effect of time on the QoL outcomes, no significant differences were found in the change of the QoL index over time between the PVP and HoLEP groups during the 60-month follow-up period (Supplementary Fig. S2). The improvement in all outcomes parameters, including total I-PSS, VSS, SSS, the maximum flow rate (*Q*_max_), PVR, and bladder voiding efficiency (BVE), was maintained during the entire follow-up period after PVP or HoLEP, except for *Q*_max_ at 60 months after PVP. However, the values of VSS and SSS starting from 36 months after the PVP were increased compared with those at 12 months after surgery, although their decrease was sustained up to 60 months after surgery compared with that at baseline. Meanwhile, the values of VSS starting from 24 months after the HoLEP were increased compared with those at 12 months after surgery, whereas those of SSS at 24, 36, 48 and 60 months after surgery were not different from 12 months postoperatively. The *Q*_max_ values in both the PVP and HoLEP groups were deteriorated starting from 24 months compared with those at 12 months after surgery. However, the increase in the *Q*_max_ value at all follow visits after the HoLEP was maintained up to 60 months compared with that at baseline, whereas the *Q*_max_ value at 60 months after the PVP was decreased to the baseline level. The incidence of transient urinary incontinence after HoLEP was higher than that after PVP (Table 2). Repeated BPH surgeries because of the regrowth of prostatic adenoma were performed for 12 patients in the PVP group but for none in the HoLEP group.

**Table 2:** Comparison of the postoperative complications between PVP and HoLEP.

	PVP	HoLEP	Clavien-Dindo classification
Transfusion	0 (0.0)	6 (0.8)	II
Recatheterization*	3 (0.7)	44 (5.8)	II
Transient dysuria	35 (8.0)	56 (7.4)	I
Urge urinary incontinence*	6 (1.4)	38 (5.0)	II
Stress urinary incontinence*	1 (0.2)	175 (23.2)	I
Urethral stricture*	2 (0.5)	18 (2.4)	IIIa
Bladder neck contracture	1 (0.2)	7 (0.9)	IIIa
Repeated BPH surgery*	12 (2.7)	0 (0.0)	IIIa

PVP = photoselective vaporization of the prostate; HoLEP = holmium laser enucleation of the prostate; BPH = benign prostatic hyperplasia.

Asterisk (*) indicates that there was a statistically significant difference. (Chisquare test or Fisher’s exact test, p < 0.05).

### Comparison of the postoperative changes in outcome parameters between PVP and HoLEP to that at the baseline

**Figure 2:**
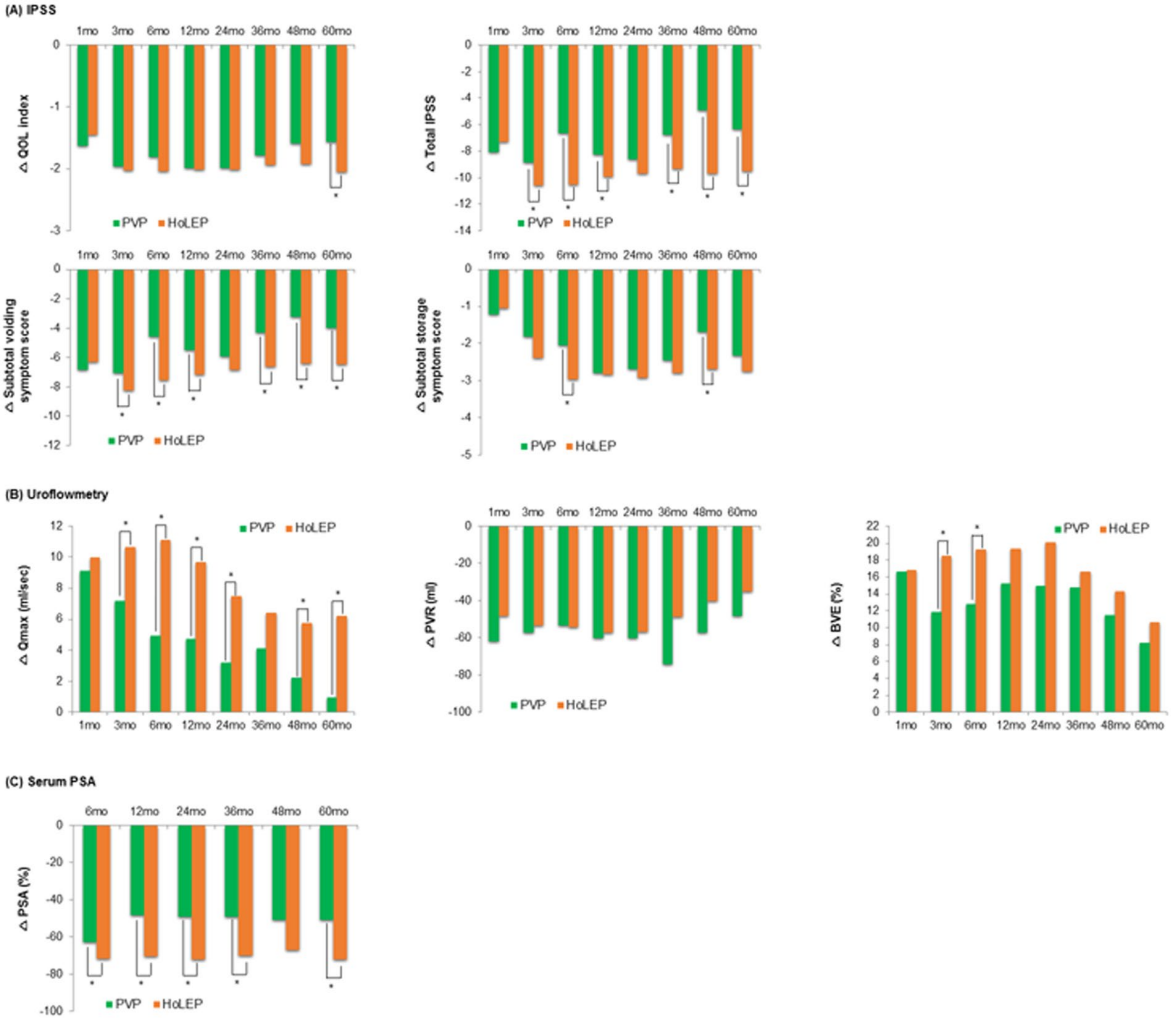
Serial changes in the outcome values at each follow-up visit after PVP and HoLEP compared with that at the baseline value. The asterisk (*) indicates a significant difference between PVP and HoLEP (independent t-test, p < 0.05).

In terms of postoperative changes from the baseline in the QoL index, no significant differences were found between the groups during the 48-month follow-up period after surgery. However, the degree of reduction in the QoL index at 60 months after HoLEP was greater than that after PVP (Fig. 2). The percentages of patients with QoL improvement were 54.0%, 52.2%, and 43.1% at 1-, 3- and 5-years after PVP, and 57.7%, 53.7%, and 55.3% at 1-, 3- and 5-years after HoLEP, respectively (Supplementary Table S1).

The degree of reduction in the total I-PSS and VSS during the entire follow-up period after HoLEP was greater than that after PVP, except for that at one month and 24 months after the surgeries (Fig. 2). Additionally, the degree of increase in *Q*_max_ during the entire follow-up period after HoLEP was greater than that after PVP, except for that at one month and 36 months after the surgeries (Fig. 2). However, no significant differences were found in the postoperative changes in the other outcome parameters (SSS, PVR, and BVE) compared with that at baseline between the two groups throughout the entire follow-up period after surgery. The degree of decrease in the serum PSA level at each follow-up visit after HoLEP was generally greater than that after PVP.

### Influential factor of QoL improvement after PVP or HoLEP

According to logistic regression (LR) analyses, univariate analysis showed that the serum PSA level, preoperative prostate volume, baseline SSS, BCI, BOOI on baseline urodynamic study (UDS), operation time, and energy applied during surgery were associated with QoL improvement at one year after surgery. Multivariate analysis revealed that lower baseline SSS and higher BOOI were independent factors influencing QoL improvement at the short-term follow-up visit after surgery (Table 3). In terms of influential factors of QoL improvement at the mid-term follow-up visit, the univariate model showed that the preoperative prostate volume, BOOI on baseline UDS, operation time, and energy applied during surgery were associated with QoL improvement at three years after surgery. However, on multivariate analysis, no independent factor influenced QoL improvement at that time (Table 3). Regarding influential factors of QoL improvement at the long-term follow-up visit, univariate analysis revealed no factor influencing QoL improvement at five years after surgery. The surgical methods were not associated with QoL improvement at any time point of follow-up after surgery (Table 3).

**Table 3:** Results of univariate and multivariate logistic regression for ‘QoL improvement.

	1 yr				3 yr				5 yr			
	Univariate		Multivariate		Univariate		Multivariate		Univariate		Multivariate	
	P	OR (95% CI)	P	OR (95% CI)	P	OR (95% CI)	P	OR (95% CI)	P	OR (95% CI)	P	OR (95% CI)
Age	0.099	0.978 (0.952–1.004)			0.926	1.001 (0.973–1.030)			0.868	0.997 (0.960–1.035)		
BMI	0.115	0.953 (0.898–1.012)			0.879	1.006 (0.933–1.084)			0.194	0.941 (0.859–1.031)		
HTN	0.453	0.870 (0.604–1.252)			0.819	1.052 (0.680–1.628)			0.978	0.992 (0.577–1.707)		
DM	0.380	0.810 (0.506–1.297)			0.223	0.679 (0.365–1.265)			0.629	1.238 (0.521–2.944)		
PSA	0.016^*^	1.055 (1.010–1.103)			0.970	0.999 (0.968–1.032)			0.063	1.065 (0.997–1.138)		
TPV	0.009^*^	1.010 (1.003–1.018)			0.016^*^	1.011 (1.002–1.019)			0.866	1.001 (0.991–1.011)		
IPSS												
VSS	0.193	0.976 (0.940–1.013)			0.120	0.967 (0.926–1.009)			0.249	0.968 (0.915–1.023)		
SSS	0.003^*^	0.918 (0.867–0.971)	0.040^*^	0.936 (0879–0.997)	0.133	0.952 (0.893–1.015)			0.053	0.925 (0.856–1.001)		
QoL	0.621	0.953 (0.787–1.154)			0.531	1.071 (0.864–1.328)			0.078	1.289 (0.972–1.708)		
*Q*_max_	0.649	1.011 (0.965–1.059)			0.614	1.015 (0.958–1.076)			0.614	1.018 (0.951–1.089)		
PVR	0.117	1.001 (1.000–1.003)			0.345	1.001 (0.999–1.004)			0.098	1.003 (0.999–1.007)		
BVE	0.681	0.844 (0.377–1.891)			0.631	0.771 (0.266–2.233)			0.275	0.447 (0.105–1.897)		
FDV	0.929	1.000 (0.998–1.002)			0.770	1.000 (0.997–1.002)			0.881	1.000 (0.997–1.004)		
MCC	0.432	1.001 (0.999–1.002)			0.691	1.000 (0.998–1.002)			0.903	1.000 (0.998–1.003)		
IDC	0.389	1.186 (0.804–1.749)			0.474	0.893 (0.519–1.356)			0.666	0.877 (0.484–1.590)		
BOOI	<0.001^*^	1.014 (1.007–1.022)	0.001^*^	1.015 (1.006–1.023)	0.011^*^	1.011 (1.003–1.020)			0.322	1.005 (0.995–1.016)		
BCI	0.017^*^	1.009 (1.002–1.016)			0.231	1.005 (0.997–1.014)			0.540	0.997 (0.986–1.007)		
Surgical method	0.426	1.160 (0.805–1.673)			0.794	1.060 (0.685–1.639)			0.089	1.633 (0.929–2.872)		
Operation time	0.003^*^	1.009 (1.003–1.015)			0.007^*^	1.008 (1.002–1.014)			0.951	1.000 (0.994–1.006)		
Used energy	0.007^*^	1.004 (1.001–1.007)			0.017^*^	1.004 (1.001–1.007)			0.946	1.000 (0.997–1.003)		

Note: BMI-body mass index, HTN-hypertension, DM-diabetes mellitus, PSA-prostate specific antigen, TPV- total prostatic volume, I-PSS-international prostatic symptom score, VSS-voiding symptom score, SSS-storage symptom score, QoL-quality of life, *Q*_max_-peak flow rate, PVR- postvoid residual urine volume, BVE-bladder voiding efficiency, FDV-first desire to void, MCC-maximum cystometric capacity, IDC-involuntary detrusor contractility, BOOI- bladder outlet obstruction index, BCI-bladder contractility index.

Asterisk (*) indicates that there was a significant difference (Univariate or multivariate logistic regression, P < 0.05).

## Discussion

Because LUTS strongly influences QoL negatively^2,4^, one of the goals for the treatment of BPH was focused on improving QoL^16^. Laser surgery for BPH has been expected to be alternatives to the gold standard of BPH surgery (TURP or open prostatectomy) because, compared with TURP, it showed comparable efficacy and lower postoperative morbidity as mentioned above^11,17^. Specifically, Xue *et al*. reported that PVP provided equivalent efficacy and fewer bleeding complications for 3 years after surgery, compared with TURP^18^. HoLEP, an enucleation surgery for BPH, was reported to have similar efficacy and lower perioperative complications with a significant level of evidence, than TURP^8^. However, there has been a scarcity of studies mainly focusing on QoL improvement after laser prostatectomy. Because the treatment outcomes of BPH surgery greatly affect patients’ QoL, this study may extend the current knowledge regarding them. The results of our study are summarized as follows:

1. Postoperative improvement of QoL was maintained up to the long-term follow-up period after PVP or HoLEP.
2. No significant differences were found between the two groups in postoperative changes from baseline of the QoL index during the 48-month follow-up period after surgery. However, the degree of improvement in QoL at 60 months after HoLEP was greater than that after PVP.
3. Lower baseline SSS and higher BOOI were independent factors influencing QoL improvement at the short-term follow-up visit after surgery. However, because the follow-up duration was longer, no independent factor influenced QoL improvement at the mid- and long-term follow-up visits after surgery.

Our study has shown that the postoperative improvement of QoL was maintained up to the long-term follow-up period, irrespective of the type of laser surgery. In agreement with our results, Xue *et al*. reported that the postoperative improvement of QoL was sustained up to 36 months after TURP or PVP-120 W-HPS^18^. Additionally, Gilling *et al*. compared the surgical outcomes at 1, 3, 6, 12, 24 and 92 months between TURP and HoLEP^19^. According to their results, the postoperative improvement of QoL was maintained up to 92 months after TURP and HoLEP, without a difference between the two surgeries^19^. Meanwhile, although laser surgery is expected to have a positive effect on the postoperative improvement of QoL in men with BPH, few studies have focused on the comparative analysis of serial long-term treatment outcomes for QoL between PVP and HoLEP. Previously, a minority of studies showed no difference in the postoperative improvement of QoL at one year after surgery between PVP using 120 W HPS and HoLEP^14,15^. Interestingly, our study with long-term follow-up data showed that the degree of improvement in the QoL index at 60-month follow-up visits after HoLEP might be superior to that after PVP, perhaps in part because of a difference in the long-term durability of surgical outcomes. This result could be supported by our finding that the degree of improvement in voiding symptoms, peak flow rate or reduction in the serum PSA level at the long-term follow-up visits after HoLEP was higher than that after PVP. In accordance with these findings, recent literature by Hermann *et al*. noted that transurethral endoscopic enucleation of the prostate, such as HoLEP, offer some advantages in terms of morbidity^8,13^. However, further long-term follow-up studies comparing PVP using the 180 W XPS laser and HoLEP are necessary to draw a solid conclusion because PVP using the 180 W XPS laser has recently been performed in men with BPH.

Interestingly, according to our data, the degree of improvement in voiding symptoms during the early postoperative period (1 to 3 months) after PVP or HoLEP was much greater than that in storage symptoms. Thus, storage symptoms appear to improve gradually with time after surgery, whereas voiding symptoms do dramatically from the immediate postoperative period after surgery. This may be attributed to an irritative effect of laser vaporization despite enucleation during laser prostatectomy. Thus, laser energy applied during laser prostatectomy might be beneficial with respect to hemostasis, but some patients could pay the price of postoperative storage or irritative symptoms in the early postoperative period. The irritative effect may have impacts on the surgical outcome for storage symptoms in the early postoperative period. In accordance with this, Cindolo *et al*. showed that transient storage symptoms were developed more frequently after GreenLight enucleation of the prostate than after standard PVP or anatomic PVP^20^. They suggested that this might be due to microinjury of capsules and per-capsule innervation caused by more coagulation in the capsular bleeding spot in GreenLight enucleation of the prostate^20^.

A factor to be considered when evaluating the surgical outcomes of PVP or HoLEP is the surgeon’s expertise. Particularly regarding the learning curve of HoLEP, some literature has shown that HoLEP has a steep learning curve with a stationary state at 20–50 cases^21,22^. Accordingly, when the learning curve for the efficiency of HoLEP was assessed by enucleation efficiency (a ratio of retrieved tissue weight/enucleation time) in our study, the enucleation efficiency appeared to be stationary after approximately 50 cases (Supplementary Fig. S1B). According to a recent study by Castellan *et al*. that analyzed a learning curve of PVP using the 180 W XPS laser, surgeons with greater experience in endoscopic procedure showed a lower rate of early complications and greater evolution in lasing time/operation time ratio than those with lesser experience^23^. However, there was no significant difference in the functional outcomes at 6 months after surgery between the groups^23^. In the present study, when the learning curve for the efficiency of PVP was assessed by the ratio of the removed prostate volume/operation time, the PVP efficiency seemed to be stationary even in the first case. Furthermore, the PVP efficiency was further evolved after approximately 150 cases. The cause might be that the surgeon (HS) in our study had sufficient experience in endoscopic surgery for BPH. However, it is difficult to directly compare the studies because of differences in the baseline characteristics and study populations, as well as in definitions of learning curve.

To our knowledge, this is the first study that reported the factors influencing the postoperative improvement of QoL serially at the short-term, mid-term, and long-term follow-up visits after PVP or HoLEP. Our LR analyses showed that a lower baseline SSS and a higher BOOI were independent factors influencing postoperative QoL improvement at the short-term follow-up visit after PVP or HoLEP. Given that BPH surgeries such as PVP and HoLEP have been designed to relieve BOO, it may be reasonable to assume that patients with a higher BOOI have a higher probability of improvement in QoL after surgery. In accordance with our result, Ryoo *et al*. showed that a BOOI greater than 40 was a predictor of treatment success, including QoL at six months after HoLEP^24^. Additionally, the patients with less severe storage symptoms before surgery appear to have a higher possibility of postoperative improvement in QoL, perhaps because, as our data showed, the lower baseline SSS was significantly correlated with a lower SSS and a lower QoL index at one year after PVP or HoLEP (Spearman’s correlation coefficient = 0.390 and 0.253, respectively). Interestingly, based on univariate analysis, several factors, including a higher BOOI and preoperative prostate volume, were associated with postoperative QoL improvement at the mid-term follow-up visit, no independent factor of QoL improvement was found on multivariate analysis. Furthermore, no influential factor of QoL improvement was found at the long-term follow-up visit, even on univariate analysis. Thus, postoperative QoL improvement at the short-term follow-up period after PVP or HoLEP appears to be maintained up to five years, without any specific factor influencing its improvement at the mid-term or long-term follow-up visit.

There are a few limitations in our study. First, because our study was retrospective, there were differences in a few baseline parameters between the two groups. Second, we used PVP using 120 W-HPS; however, in 2010, PVP using 180 W-XPS was introduced. In the future, long-term follow-up studies comparing PVP using the 180 W XPS laser and HoLEP are necessary to validate our result, although the long-term follow-up data of PVP using 180 W-GreenLight XPS might still be limited. Nevertheless, our data have some clinical implications to understand the outcomes of QoL-related LUTS after laser prostatectomy, such as PVP or HoLEP. Our results may help effectively counsel patients about expectations for two representative laser prostatectomies or surgical outcomes for QoL related to LUTS. Additionally, our results may be used to counsel patients on which ones can benefit the most from PVP or HoLEP in terms of QoL-related LUTS, particularly at the short-term follow-up period after surgery.

In conclusion, our data confirm that both PVP and HoLEP have durable efficacy in QoL improvement throughout the five-year follow-up period. HoLEP might provide more improvement in QoL at the long-term follow-up point than PVP. A lower baseline SSS and a higher BOOI appear to independently influence QoL improvement at the short-term follow-up visit after PVP or HoLEP. When the follow-up duration is longer, no factor appears to influence QoL improvement independently at the mid- and long-term follow-up visits. Subsequent prospectively controlled comparative studies with a larger study population and a long-term follow-up on PVP using 180 W GreenLight XPS and HoLEP would be needed to validate our findings.

## Materials and Methods

### Study population, data and design

This study was approved by the Institutional Review Board at both Seoul National University and Seoul Metropolitan Government Seoul National University Boramae Medical Center. All methods in this study were carried out in accordance with relevant guidelines and regulations. The need for written informed consent was waived because of the retrospective nature of this study. We retrospectively reviewed the data of 1,569 patients who had undergone PVP-120 W-HPS (n = 564) or HoLEP (n = 1,005) because their LUTS/BPH was unresponsive to medications from January 2008 to March 2014 at our institution. We excluded 320 patients with previously diagnosed urethral stricture, cancer of the bladder or prostate or urethra, history of other urologic surgeries, or incomplete data. Among the remaining 1,249 patients, 1,193 patients (PVP group, n = 439; HoLEP group, n = 754) with a baseline QoL of the I-PSS of at least 2 before surgery were included in this study.

Before the surgeries, all patients received preoperative evaluations for LUTS secondary to BPH, including medical history, physical examinations, the I-PSS, urinalysis, serum PSA, transrectal ultrasound for prostate, and a multichannel UDS. A surgical method, either PVP or HoLEP, was chosen based on the surgeon’s preference. PVP was performed by a single surgeon (HS) as described in a previous study^25^. Briefly, PVP was performed using the planned vaporization-resection technique, with a 120 W GreenLight HPS laser at a setting of 80 W for vaporization-resection and 100 W for vaporization. HoLEP was performed by one of two surgeons (JSP or SJO) in the usual manner as previously mentioned^26^. Briefly, enucleation was performed mainly using three-lobe techniques with a 26 Fr resectoscope, a 550-μm laser fiber, or an 80-W holmium:YAG laser at a setting of 2 J 50 Hz or 2 J 40 Hz. Morcellation of the enucleated prostatic adenoma was performed with a morcellator. The patients received follow-up visits serially at 1, 3, 6, 12, 24, 36, 48 and 60 months after surgery. At each follow-up visit, the patients were evaluated for the I-PSS, serum PSA, and uroflowmetry.

We defined ‘QoL improvement’ as a reduction of 50% or more in the QoL index at each follow-up visit compared with that at the baseline to investigate patients with a definite effect after surgery to confirm strong factors leading to favorable outcomes. Bladder voiding efficiency (BVE) was equated as (voided volume) × 100/(voided volume + PVR).

### Statistical analysis

To compare the preoperative characteristics and surgical outcomes between PVP and HoLEP, we used independent t-test and chi-squared or Fisher’s exact test. For comparisons between baseline variables and postoperative outcome parameters, we used paired t-test. Additionally, the repeated-measures ANOVA test was performed to adjust for the impact of time on the QoL outcomes. To identify the factors influencing the ‘QoL improvement’ at the short-term (one year after surgery), mid-term (three years after surgery), and long-term (five years after surgery), we applied LR analyses. Variables with p < 0.05 in the univariate LR were included in multiple LR. p < 0.05 was considered as statistically significant in all analyses. Statistical analyses were performed using IBM SPSS version 21.0.

## Supplementary information

Supplementary information

## Data Availability

The analyzed data sets of this study can be reasonably requested from the corresponding author.
