# Publisher Correction: Quality of life after photo-selective vaporization and holmium-laser enucleation of the prostate: 5-year outcomes

**DOI:** 10.1038/s41598-019-53162-w

**Published:** 2019-11-08

**Authors:** Inyoung Sun, Sangjun Yoo, Juhyun Park, Sung Yong Cho, Hyeon Jeong, Hwancheol Son, Seung-June Oh, Jae-Seung Paick, Min Chul Cho

**Affiliations:** 1 Department of Urology, Seoul National University College of Medicine, SMG-SNU Boramae Medical Center, Seoul, 07061 Republic of Korea; 2 Department of Urology, Seoul National University College of Medicine, Seoul National University Hospital, Seoul, 03080 Republic of Korea

Correction to: *Scientific Reports* 10.1038/s41598-019-44686-2, published online 04 June 2019

In the original version of this Article, the author Sung Yong Cho was incorrectly indexed. This error has now been corrected.

