# Supplementary material for: Quality of life after photo-selective vaporization and holmium-laser enucleation of the prostate: 5-year outcomes
Source: Sci Rep. 2019 Jun 4;9:8261. doi: 10.1038/s41598-019-44686-2 (PMC6547661; doi:10.1038/s41598-019-44686-2)

Title

Quality of life after photo-selective vaporization and holmium-laser enucleation of the prostate: 5-year outcomes

Authors

Inyoung Sun[2] Sangjun Yoo[1], Juhyun Park[1], Sung Yong Cho[1], Hyeon Jeong[1], Hwancheol Son[1,] Seung-June Oh[2], Jae-Seung Paick[2] and Min Chul Cho[1]*

Affiliations

[1] Department of Urology, Seoul National University College of Medicine, SMG-SNU Boramae Medical Center, Seoul 07061, Republic of Korea

[2]Department of Urology, Seoul National University College of Medicine, Seoul National University Hospital, Seoul 03080, Republic of Korea

# Correspondence:

Associate Professor Min Chul Cho, M.D., Ph.D.

Department of Urology, Seoul National University Boramae Medical Center,

5 Gil 20, Boramae-Road, Dongjak-Gu, Seoul, South Korea, Zip code: 07061

Tel.+82-2-870-2393

Fax.+82-2-870-2826

E-mail: cmc1206@empal.com

Supplementary Table S1. Percentage of patients with QoL improvement at 1, 3, 6, 12, 24, 36, 48, and 60 months after the PVP or HoLEP

	PVP	HoLEP
Period after surgery		
1 month	46.0%	41.5%
3 months	55.8%	59.1%
6 months	49.7%	59.0%
12 months	54.0%	57.7%
24 months	55.2%	53.1%
36 months	52.2%	53.7%
48 months	45.6%	52.9%
60 months	43.1%	55.3%

Supplementary Figure S1. Lerning curves of photoselective vaporization of the prostate (PVP) or holmium laser enucleation of the prostate (HoLEP)

A. PVP learning curve (Reduced prostate volume (mL) / Operation time (min))

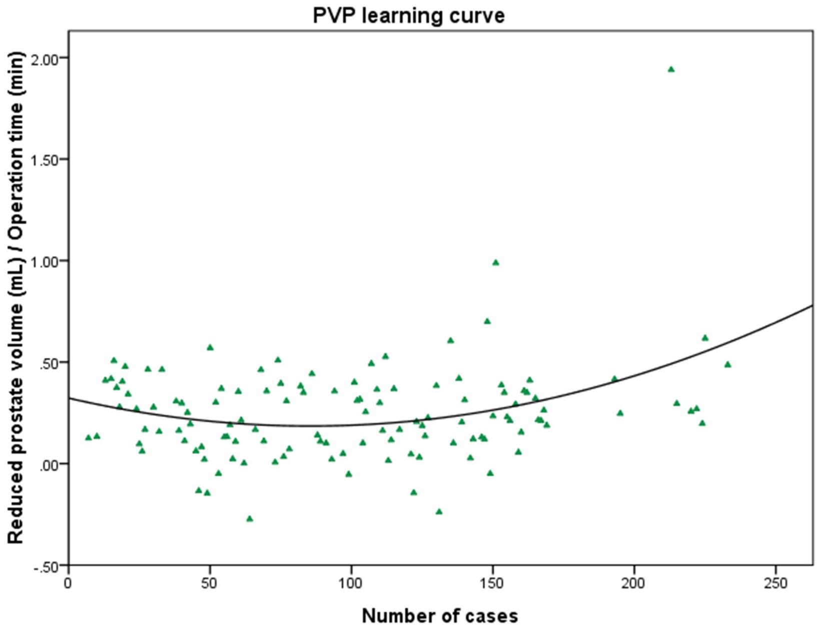

B. HoLEP learning curve (retrieved tissue weight (g) / Enucleation time (min))

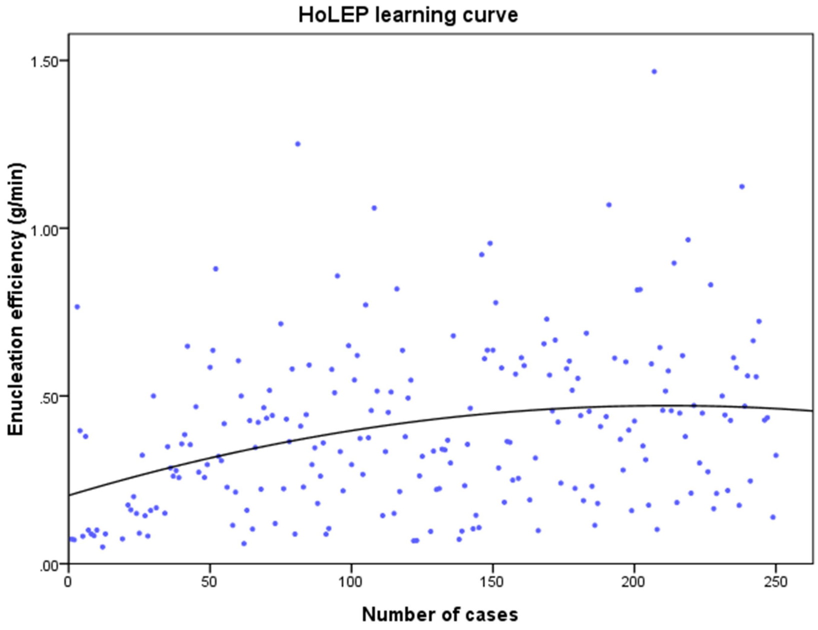

Supplementary Figure S2. Serial postoperative QoL outcomes at 12- (short-term), 36- (mid-term) and 60-months (long-term) after PVP or HoLEP. The repeated measures ANOVA test was performed to adjust for the effect of time on the QoL outcomes. Asterisk (*) indicates that at each follow up visit, there were significant differences from the value at baseline ($p < 0.05$). QoL = quality of life, PVP = photoselective vaporization of the prostate (PVP), HoLEP = holmium laser enucleation of the prostate.

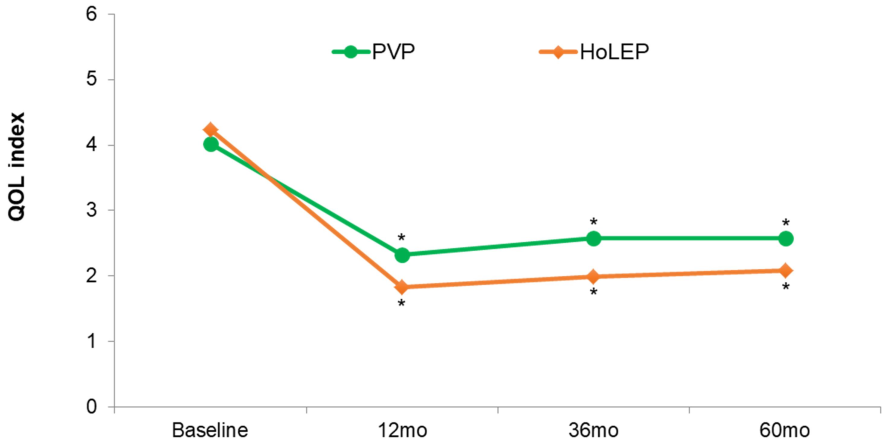

Supplement: Supplementary file 1 — Supplementary information [file 41598_2019_44686_MOESM1_ESM.pdf]
